# Transcriptional signatures of the cortical morphometric similarity network gradient in left temporal lobe epilepsy with different seizure symptoms

**DOI:** 10.3389/fnins.2026.1833695

**Published:** 2026-06-03

**Authors:** Kanlin Lin, Taipeng Zeng, Pan Zhang, Hui Li, Pengfan Yang, Zhifeng Huang, Nuozhen Chen, Xiaoyang Wang, Liyuan Fu, Shangwen Xu

**Affiliations:** 1 Fuzong Teaching Hospital of Fujian University of Traditional Chinese Medicine, Fuzhou, China; 2 900th Hospital of PLA Joint Logistic Support Force, Fuzhou, China

**Keywords:** focal to bilateral tonic–clonic seizures, gene expression, gradient, morphometric similarity network, temporal lobe epilepsy

## Abstract

**Background:**

Temporal lobe epilepsy (TLE) manifests with diverse seizure symptoms, including focal to bilateral tonic—clonic seizures (FBTCS), linked to widespread brain network disruptions. The role of cortical morphometric similarity (MS) network gradients and their relationship with gene expression in TLE remains unclear.

**Methods:**

We studied MS network gradient abnormalities through group comparisons among 87 left TLE patients (48 FBTCS−, 39 FBTCS+) and 63 healthy controls (HC). In addition, partial least squares (PLS) regression analysis was performed to investigate the association between gradient changes and whole-brain gene expression in left FBTCS+ TLE patients.

**Results:**

FBTCS+ patients showed significant reductions in the principal MS network gradient within default mode network (DMN) regions compared to healthy controls, while FBTCS− patients exhibited no such abnormalities. Gradient alterations in FBTCS+ were linked to whole-brain expression of genes involved in neurobiological pathways, cell types, and cortical layers.

**Conclusion:**

FBTCS+ TLE is associated with distinct MS network gradient alterations, which may reflect underlying molecular mechanisms contributing to structural changes linked to severe seizure symptoms.

## 1 Introduction

Temporal lobe epilepsy (TLE) is one of the most common forms of focal epilepsy (Yakovleva et al., 2022; Tabibian et al., 2023), which is characterized by recurrent seizures and impairments in cognitive and emotional functions (Zaitsev and Khazipov, 2023). These deficits significantly reduce patients’ quality of life and social functioning. Focal to bilateral tonic–clonic seizures (FBTCS) represent a common and clinically consequential seizure subtype in TLE, characterized by a high frequency of occurrence and significant clinical impact. FBTCS can rapidly spread to multiple regions of the brain, with progression to bilateral tonic–clonic convulsions and impaired consciousness, along with epilepsy-related injuries, sudden unexpected death in epilepsy, and poor surgical outcomes (Blumenfeld et al., 2009; Janszky et al., 2005). Moreover, patients with FBTCS often exhibit greater cognitive impairment, particularly in memory, further compromising quality of life (Prevey et al., 1998). Although the pathophysiological mechanisms of TLE have been widely studied, the specific neural basis and mechanisms of FBTCS in TLE remain poorly understood, further highlighting the necessity for in-depth investigation (Ge et al., 2024).

Extensive research utilizing magnetic resonance imaging (MRI) has explored specific structural and functional brain alterations in patients with TLE (Crow et al., 2023; Ellsay and Winston, 2024). Studies have shown that TLE patients exhibit structural and functional brain alterations, including reduced gray matter volume, cortical thinning, impaired white matter integrity, and disrupted connectivity in networks like the default mode network (DMN) and limbic system, which are linked to cognitive and emotional deficits (Pizzanelli et al., 2022; Fonseca et al., 2023; Mueller et al., 2012; Leyden et al., 2015). These findings suggest that structural and functional abnormalities in TLE may reflect disruptions in neural network integration (Burianová et al., 2017). However, the specific brain connectivity patterns and underlying molecular mechanisms associated with different seizure symptoms in TLE patients remain incompletely understood.

Morphometric similarity (MS) gradients characterize continuous variations in cortical morphometric organization across the brain, providing insights into the underlying mechanisms of neural development and the hierarchical organization of brain networks (Sadikov et al., 2025; Yin et al., 2024; Zeng et al., 2026; Yang et al., 2021). Conceptually, the principal gradient delineates a macroscopic spatial axis that typically transitions from primary unimodal sensory-motor cortices to higher-order transmodal areas, such as the default mode network. This continuous spatial organization serves as a foundational coordinate system for understanding how localized structural vulnerabilities propagate along the cortical hierarchy, ultimately affecting large-scale brain networks (Yang et al., 2021; Li et al., 2021). Over recent years, the combination of morphometric networks, gradient mapping, and transcriptomic enrichment has evolved into a relatively mature methodological framework for investigating complex neuropsychiatric conditions (Xue et al., 2023). In the specific context of epilepsy, this framework has been successfully employed to capture system-wide network reconfigurations. For instance, prior studies have demonstrated that focal epicenters in epilepsy can induce widespread network alterations, often manifesting as macroscopic hierarchical disruptions such as gradient contractions or spatial shifts, which correlate with clinical severity and cognitive dysfunction (Royer et al., 2023; Zhang et al., 2023). These prior applications highlight the utility of the gradient framework in translating regional imaging abnormalities into a broader understanding of epilepsy-related network pathology (Xie et al., 2024; Lu et al., 2025). MS networks have been shown to align with spatial patterns of gene expression, thereby providing a useful framework for linking macroscale brain organization to underlying transcriptional architecture (Qu et al., 2024; Yao et al., 2024). Previous studies have revealed widespread alterations in brain regions such as the frontal lobe in mTLE patients, and enrichment analysis has highlighted pathways associated with neurodevelopment and neurodegenerative diseases, offering a novel perspective on the relationship between macroscopic morphometric measures and transcriptional profiles (Lu et al., 2025). However, while gradient analysis has been applied to various neurological disorders to uncover functional connectivity abnormalities, its specific application in understanding seizure generalization, such as TLE with FBTCS, remains limited (Lucas et al., 2023). Specifically, for TLE with FBTCS, the MS network gradient may capture large-scale cortical coordination in morphometric architecture and provide distinct imaging phenotypes associated with seizure propagation.

Recent work has highlighted the contribution of genetic architecture to the spatial organization of brain networks (Zhang et al., 2026). Tools like the Allen Human Brain Atlas (AHBA) enable the integration of gene expression data with large-scale brain changes, offering a deeper understanding of how molecular-level disruptions manifest in neurological disorders (Arnatkeviciute et al., 2019). In TLE, imaging-transcriptomics approaches allow researchers to explore the links between genetic expression and structural or functional brain abnormalities, providing new perspectives on the disorder’s underlying mechanisms (Qin et al., 2024).

We hypothesized that FBTCS-related seizure generalization would be associated with alterations in the large-scale organization of cortical morphometric similarity networks. In this study, we utilized MS networks and diffusion map embedding to construct the principal MS gradient and compared the organizational profiles of MS gradients among FBTCS+, FBTCS− TLE patients, and healthy controls (HC). Subsequently, partial least squares (PLS) regression was applied to link MS gradient changes with gene expression patterns, aiming to identify key genes associated with TLE heterogeneity. Additionally, multiple enrichment analyses were conducted to connect gene expression with molecular pathways, cell types, and brain structural features, providing a multi-level exploration of the neurobiological differences and potential mechanisms underlying the two patient groups.

## 2 Materials and methods

### 2.1 Subjects

The study received ethical approval from the Ethics Committee of the 900th Hospital of PLA Joint Logistic Support Force (Approval Number: 2019-005). All participants and their families provided written informed consent in compliance with the principles outlined in the Declaration of Helsinki.

A total of 87 left TLE patients and 63 age and sex-matched healthy controls (HC) were included. Patients were categorized into two groups, FBTCS+ (*n* = 39), comprising individuals with bilateral tonic–clonic seizures, and FBTCS− (*n* = 48), encompassing those without such seizures. Specifically, FBTCS− patients had never experienced generalized tonic–clonic seizures (GTCS) in their lifetime, whereas FBTCS+ patients had experienced one or more such events. All patients met the following inclusion criteria: (1) right-handedness; and (2) a diagnosis of TLE based on the 2017 International League Against Epilepsy (ILAE) criteria (Wirrell et al., 2022). Specifically, for patients with left TLE, the diagnosis was established by the Epilepsy Center physicians based on video-electroencephalography, clinical seizure symptomatology, and neuroimaging findings.

The exclusion criteria for patients with TLE were as follows: (1) age under 16 years; (2) epileptogenic focus located outside the temporal lobe; (3) history of traumatic brain injury or neurosurgical procedures; (4) structural abnormalities unrelated to TLE, such as tumors, vascular malformations, or extensive cortical malformations; (5) history of neurological or psychiatric disorders, or other severe systemic diseases; and (6) contraindications for MRI examination.

### 2.2 Image acquisition and preprocessing

MRI data for all participants were collected using a 3.0 T Siemens Magnetom Trio Tim superconducting magnetic resonance system. Participants were scanned in a supine position with earplugs and foam padding to reduce head motion. During the scan, they were instructed to keep their eyes closed, remain still, and refrain from engaging in any conscious cognitive activities. Anatomical imaging was performed using a 3D gradient-echo sequence with the following parameters: TR = 1900 ms, TE = 2.5 ms, flip angle = 9°, bandwidth = 170 Hz/pixel, slice thickness = 1.0 mm, 160 slices, and a field of view (FOV) of 256 × 256 mm (Tabibian et al., 2023). The total acquisition time for this sequence was 4 min and 26 s. High-resolution 3D T1-weighted images were processed using FreeSurfer (v8.0.0) to reconstruct cortical surfaces. The preprocessing pipeline included skull stripping, tissue segmentation, surface reconstruction, metric calculation, and spherical normalization parameter estimation. Quality control (QC) involved visual inspection of skull stripping and segmentation, correction of surface reconstruction errors, and exclusion of outliers with metrics deviating beyond two standard deviations from the group mean. Importantly, no participants were excluded based on this criterion, as all metrics fell within the acceptable range.

### 2.3 Construction of MS gradients

The DK atlas was first divided into 68 cortical regions and further segmented into 308 spatially adjacent areas (Seidlitz et al., 2018; Desikan et al., 2006). These regions were subsequently mapped onto the cortical surface of each participant. For each region, five morphometric features were extracted from T1-weighted images. These features included surface area, cortical thickness, gray matter volume, intrinsic curvature, and mean curvature. To account for differences in the distributions of feature values, z-score normalization was applied to each feature vector. Pairwise Pearson correlation analysis was performed on the normalized feature vectors to evaluate the similarity between cortical regions. This process generated a 308 × 308 morphometric similarity network for each participant, with no thresholding applied. The average weighted correlation coefficient between each region and all other regions was then calculated to quantify MS network connectivity and intensity. An affinity matrix was generated from the MS network using a Gaussian kernel. Diffusion map embedding, implemented using Brain Space (v0.1.4), was applied as a nonlinear dimensionality reduction method to decompose the affinity matrix into gradient components that represent the principal axes of morphometric similarity. The principal gradient, capturing the largest variance, was mapped onto each participant’s cortical surface for spatial visualization and group comparisons.

### 2.4 The MS gradient comparison

This study employed a general linear model (GLM) to investigate group differences in regional principal MS gradients, controlling for the effects of age and sex. Additionally, the analysis incorporated the Yeo cortical atlas, which classifies regions based on resting-state functional networks. To examine gradient changes at the network level, the mean principal MS gradient scores across all regions within each Yeo network were calculated (Yeo et al., 2011). GLM was then applied to compare the patient and control groups in terms of these network-level gradient scores, with the same covariates controlled. To ensure the robustness of statistical findings, significance was determined using different correction methods based on the level of analysis: for brain regions, a threshold of *p* < 1/n (where *n* = 308 regions) was applied, which is equivalent to saying that we expect less than one false-positive regional result per cortical map at this threshold (Lynall et al., 2010; Long et al., 2023). While for networks and classifications, the Benjamini-Hochberg false discovery rate (BH-FDR) method was used, with the threshold set at *p* < 0.05.

### 2.5 Gene expression data preprocessing

Gene expression data were obtained from the AHBA dataset,^1^ which provides transcriptomic profiles from six postmortem brains across 3,702 spatial locations (Hawrylycz et al., 2012). The data were preprocessed using the Abagen toolbox^2^ following established protocols (Markello et al., 2021). The preprocessing pipeline involved several steps: (i) mapping microarray probes to gene symbols; (ii) filtering out probes with low intensity, defined as those whose expression levels fell below the background threshold in more than 50% of samples; (iii) selecting the probe with the highest regional variation homogeneity when multiple probes targeted the same gene; (iv) assigning tissue samples to brain regions within a 2 mm Euclidean distance from the region boundary; and (v) normalizing gene expression values across samples using a scaled robust sigmoid function. Given the limited availability of right hemisphere data in the AHBA dataset, our analysis focused exclusively on the left hemisphere. As a result, a transcriptomic matrix was constructed, encompassing 152 brain regions and 15,632 genes.

### 2.6 Transcription-neuroimaging association analysis

The co-expression of genes and the influence of spatial proximity, where nearby regions exhibit more similar expression patterns, suggest that regional gene expression profiles can be effectively simplified into a small number of principal components that capture most of the variance. To investigate the spatial relationship between gene expression and t-statistic maps obtained from 152 cortical regions, we utilized PLS regression (Liu et al., 2022). The PLS regression analysis was conducted using MATLAB R2024b (MathWorks Inc., Natick, MA, United States). This method modeled the expression of 15,632 genes as predictor variables and the *t*-statistic maps as response variables. The first PLS component (PLS1) was identified as the linear combination of gene expression most strongly associated with the t-statistic maps (Abdi and Williams, 2013). To determine whether the covariance between PLS1 transcriptomic scores and the t-statistic maps exceeded random expectations, we conducted 10,000 permutation tests. Additionally, we used bootstrap resampling with 10,000 iterations to estimate the variability of each gene’s contribution to PLS1. Z-scores were calculated by dividing each gene’s regional weight by its bootstrap standard error, and genes were ranked according to their PLS1 weights. Based on these weights, significant genes were divided into two groups: PLS1 + genes and PLS1 − genes (*p* < 0.005, BH-FDR correction). The relationship between PLS1 scores and the t-statistic maps was then analyzed using Spearman rank correlation and further evaluated for spatial correlation.

### 2.7 Enrichment analyses

**Figure 1:**
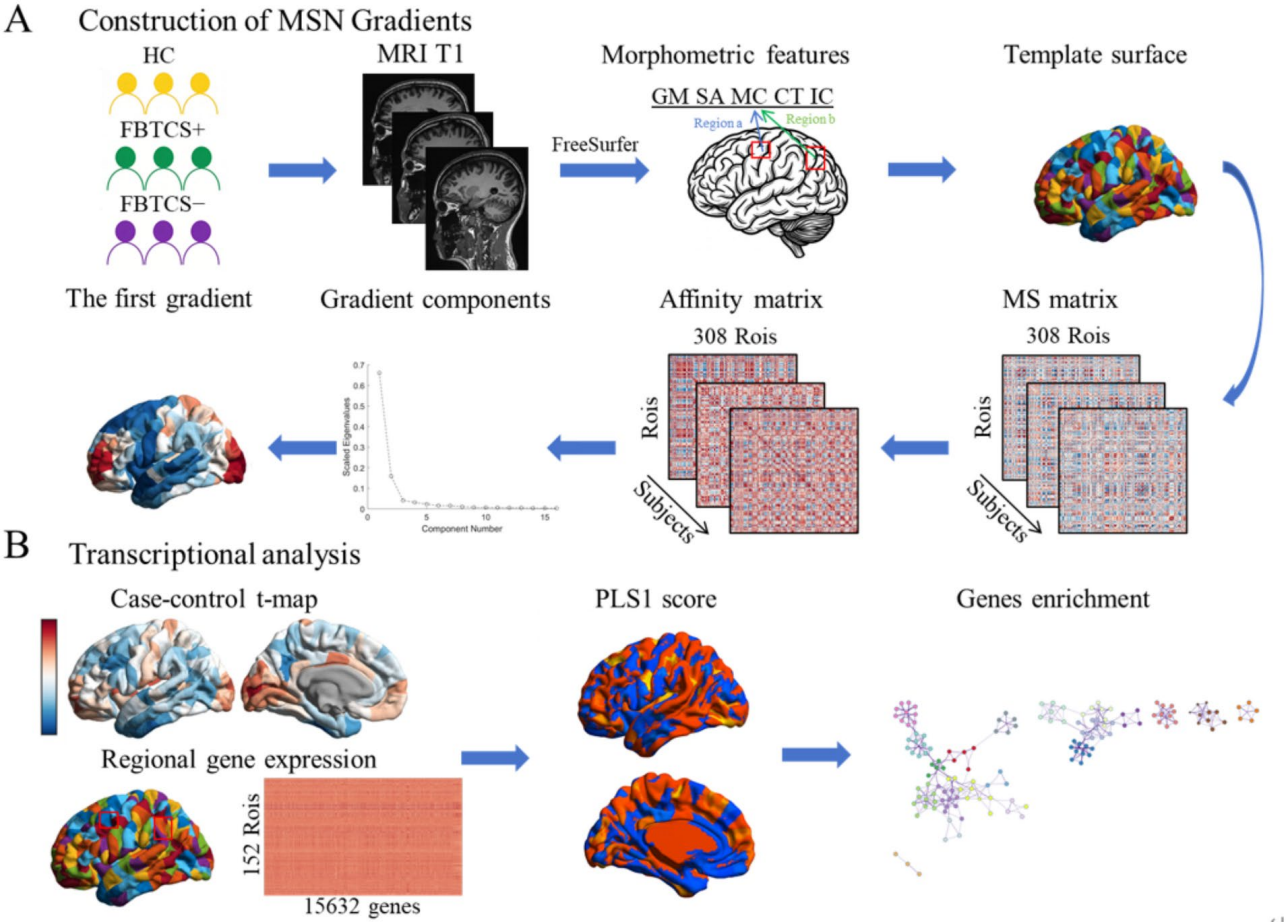
Schematic diagram of the study design. **(A)** Gradient construction process: First, morphometric features, including gray matter volume (GM), surface area (SA), cortical thickness (CT), intrinsic curvature (IC), and mean curvature (MC), are extracted from individual structural imaging data. Based on the Desikan-Killiany (DK) atlas, morphometric features of each brain region are obtained and combined into feature vectors for each region. Pearson correlation coefficients are then calculated between the feature vectors of all brain regions, resulting in a MS matrix for each subject. The MS matrix is subsequently transformed into an affinity matrix using a kernel function. Finally, diffusion embedding is applied to decompose the affinity matrix, yielding the principal gradient map for each subject. **(B)** Gene expression and transcriptomic analysis: Gene expression data for each brain region (limited to the left hemisphere) are extracted from the AHBA to construct a gene expression matrix. PLS regression is performed to associate the abnormalities in the principal morphometric gradient of FBTCS with the gene expression data. Enrichment analysis is then conducted on the significant gene list from PLS1 to identify the biological relevance of these genes. CT, cortical thickness; GM, gray matter volume; IC, intrinsic curvature; MC, mean curvature; MS, morphometric similarity; PLS, partial least squares regression; SA, surface area.

We investigated whether significant PLS+/− genes were enriched in pathways linked to TLE and other brain disorders by utilizing a gene catalog containing differential expression data from five major psychiatric conditions. For this analysis, only genes with negative weights in the first PLS component (PLS1−) were included. Functional annotation of these genes was performed using Metascape,^3^ which integrates multiple databases such as Gene Ontology (GO) and Reactome (Zhou et al., 2019). To explore cell-type-specific expression patterns, we applied the specificity index probability (pSI) package in R, which allowed us to identify the association of PLS1 − genes with distinct cell types (Dougherty et al., 2010). Cortical enrichment was further examined using marker genes derived from previous transcriptomic studies, while developmental gene expression analysis was conducted through the Cell-Type Specific Expression Analysis (CSEA) tool^4^ (Dougherty et al., 2010). All enrichment analyses used the 15,632 genes from the AHBA pipeline as the background gene set to ensure robust significance testing. All enrichment analyses were adjusted for multiple comparisons using the BH-FDR, with significance set at *p* < 0.05, Figure 1.

### 2.8 Statistical analysis

The statistical analysis was performed based on the type and distribution of the data. For continuous variables such as age, age of onset and duration, the Shapiro–Wilk test was used to assess normality. If the data followed a normal distribution, one-way analysis of variance (ANOVA) was used to compare age among the three groups (FBTCS+, FBTCS−, and HC), while an independent samples *t*-test was used to compare age of onset and duration between the FBTCS+ and FBTCS− groups. If the data did not follow a normal distribution, the Kruskal-Wallis test was applied for age comparisons and the Mann–Whitney U test for age of onset and duration comparisons. Median and interquartile ranges (Q1 ~ Q3) were calculated for continuous variables using descriptive statistics. For categorical variables such as gender, differences in distribution among the three groups were analyzed using the Chi-square test. To account for potential spatial autocorrelation in the transcriptomic data, spin permutation tests were performed to ensure the robustness of the statistical associations (Fulcher et al., 2021).

## 3 Results

### 3.1 Demographic information and clinical characteristics

No significant group differences were observed in age or sex among the FBTCS+, FBTCS−, and HC groups. A significant difference in age of onset was observed between FBTCS+ and FBTCS− patients (*Z* = −2.033, *p* = 0.042), with FBTCS+ patients showing an earlier age at seizure onset than FBTCS− patients. Additionally, there was no significant difference in disease duration between FBTCS+ and FBTCS− groups (Table 1).

**Table 1:** Demographic information and clinical characteristics.

Basic information		FBTCS+ (*n* = 39)	FBTCS− (*n* = 48)	HC (*n* = 63)	Statistic	*p* value
Age (year)		29.00 (24.00 ~ 36.00)	29.50 (25.00 ~ 39.75)	26.00 (23.00 ~ 35.00)	3.035	0.219
Gender (M/F)		20/19	22/26	28/35	0.472	0.790
Age at seizure onset (year)		13.00 (8.00 ~ 22.00)	20.00 (11.25 ~ 29.75)	—	−2.033	0.042
Duration (year)		15.00 (9.00 ~ 20.00)	10.50 (4.00 ~ 18.00)	—	1.884	0.060
Final pathology (*N*)	HS	10	13	—	—	—
	FCD	0	1	—	—	—
	Gliosis	29	34	—	—	—

FBTCS+, TLE patients with focal to bilateral tonic–clonic seizures; FBTCS−, TLE patients without focal to bilateral tonic–clonic seizures; HC, Healthy controls; F, Female; M, Male; HS, hippocampal sclerosis; FCD, focal cortical dysplasia. Data are presented as median (interquartile range) for continuous variables and counts for categorical variables. Group differences in age were assessed using the Kruskal-Wallis test; sex distribution was compared using the chi-square test; age at onset and disease duration were compared between patient groups using Mann–Whitney U test.

### 3.2 Differences in the principal MS gradient across groups

**Figure 2:**
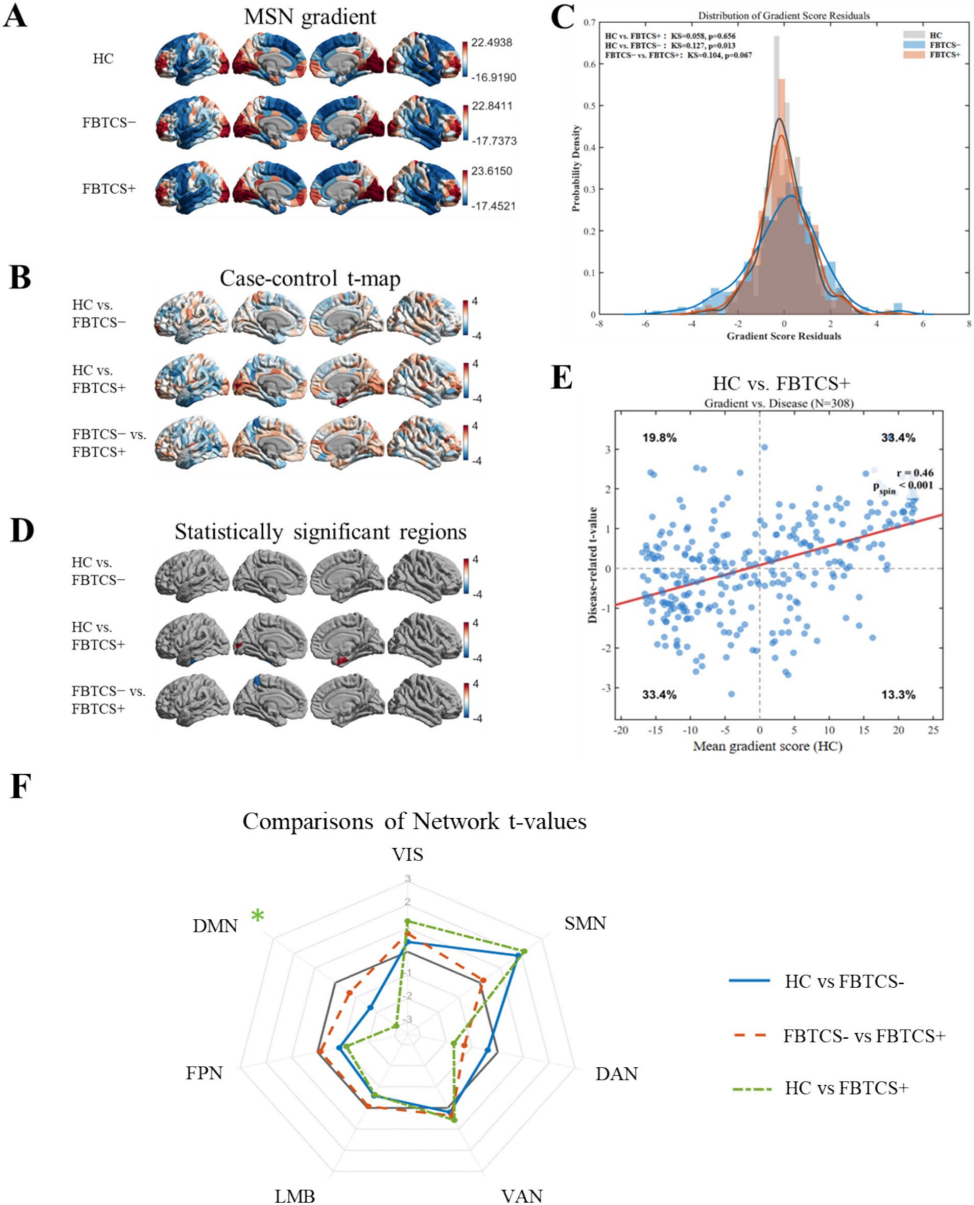
Principal MS gradients in the FBTCS+, FBTCS−, and HC groups. **(A)** Principal MS gradient patterns in TLE patients and the HC group. The motor and sensory cortices are anchored at the two extremes, while the association cortex is positioned in the middle. Regions with similar morphometric similarity profiles are shown in similar colors. **(B)** Regional statistical comparisons of morphometric profiles among healthy controls, FBTCS+ patients, and FBTCS− patients. **(C)** Distribution of mean principal MS gradient scores. **(D)** Regional statistical comparisons among healthy controls, FBTCS+ and FBTCS− patients (*p* < 1/*n*, where *n* = 308 brain regions). **(E)** Density scatter plot of the average regional MS gradient scores (x-axis) and case–control *t*-values (y-axis) in the HC group (*r* = 0.46, *p*spin < 0.001). **(F)** Absolute *t*-values of the principal MS gradient based on the Yeo7 functional networks, indicating significant differences primarily in the DMN. (* indicates FDR-corrected *p* < 0.05). BH-FDR, Benjamini–Hochberg false discovery rate; DAN, dorsal attention network; DMN, default mode network; FPN, frontoparietal network; LMB, limbic network; FBTCS, focal to bilateral tonic–clonic seizures; SMN, sensorimotor network; VAN, ventral attention network; VIS, visual network.

In our study, the principal MS gradient explained 66.1% of the variance in the MS network. To investigate individual differences, we employed a gradient alignment approach and used a GLM to compare the principal MS gradient scores among the FBTCS+, FBTCS−, and HC groups, with age and gender included as covariates in the analysis (Figures 2A,B). As illustrated in Figure 2C, after adjusting for the effects of age and gender, a significant difference in the distribution of mean principal MS gradient scores was identified between the FBTCS− group and the HC group (two-sample Kolmogorov–Smirnov test, *p* = 0.013). In contrast, no significant differences were observed between the HC group and the FBTCS+ group (*p* = 0.656) or between the FBTCS− and FBTCS+ groups (*p* = 0.067).

Regional analysis showed that, relative to HC, FBTCS+ patients exhibited decreased principal MS gradient scores in the left inferior temporal gyrus (part 3) (*p =* 0.002, *t* = −3.1614). Conversely, the principal MS gradients in the left pericalcarine cortex (part 1) (*p =* 0.001, *t* = 3.3141) and the right entorhinal cortex (part 1) (*p =* 0.003, *t* = 3.0497) were increased relative to the HC group. Additionally, in the left precuneus (part 2) (*p =* 0.002, *t* = −3.1628), the principal MS gradient was lower in FBTCS+ TLE patients compared to FBTCS− TLE patients (Figure 2D).

A positive correlation was observed between the average regional MS gradient in the HC group and the *t*-values comparing HC and FBTCS+ groups (*r* = 0.46, *p*spin < 0.001, Figure 2E), suggesting that regions located toward the extremes of the normative gradient showed greater case–control differences in FBTCS+ patients.

We also applied a previously established cortical region classification approach. According to the Yeo 7 functional network atlas, FBTCS+ patients exhibited a decreased principal MS gradient within the default mode network (FDR-corrected *p* = 0.0276, *t* = −2.9522, Figure 2F).

### 3.3 Genetic foundations of transcription-neuroimaging relationships

**Figure 3:**
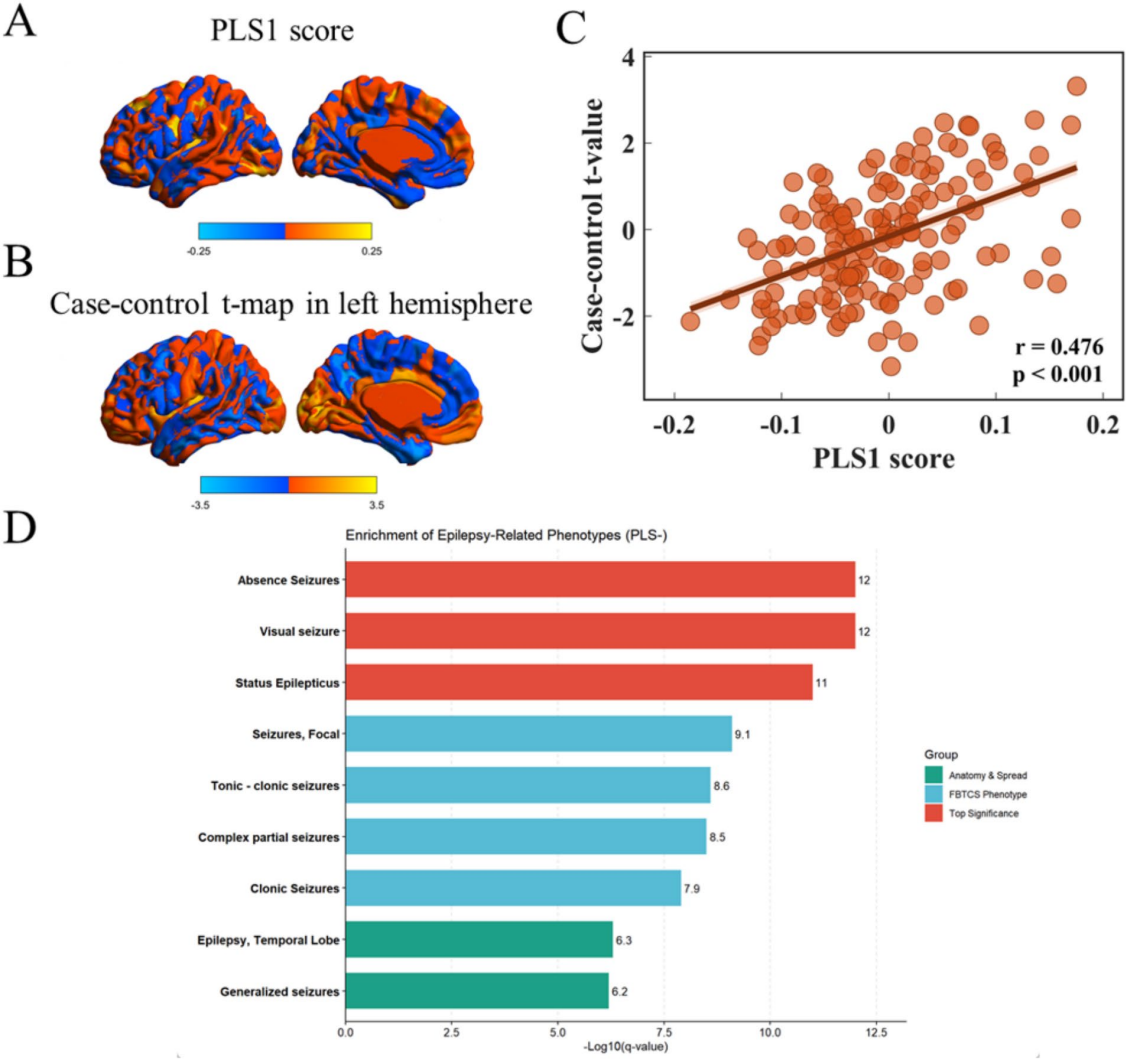
Gene expression differences between FBTCS+ TLE and HC along the principal MS gradient. **(A)** Regional map of weighted PLS1 gene expression scores in the left hemisphere. **(B)** The case–control *t*-map of the principal MS gradient scores in the left hemisphere. **(C)** A density scatter plot illustrating the relationship between regional PLS1 scores and variations in the principal MS gradient (*r* = 0.476, *p* < 0.001). **(D)** Enrichment of epilepsy-associated phenotypes based on PLS1 analysis.

The brain gene expression matrix was obtained from the AHBA database. The distribution of PLS1 scores revealed an anterior-to-posterior gradient of gene expression across the left hemisphere (Figure 3A). To further investigate this gradient, the brain gene expression matrix (152 regions×15,632 genes) was used in a PLS regression analysis to identify gene expression patterns associated with the anatomical distribution of the principal MS gradient differences. Between HC and FBTCS+ groups (Figure 3B), PLS1 accounted for 29.8% of the variance in the principal MS gradient differences between cases and controls, a value significantly higher than expected by chance (spin test, *p*spin < 0.001). The PLS1 score map also showed a positive correlation with the case–control *t*-map (*r* = 0.47, *p*spin < 0.001, Figure 3C). In contrast, no significant correlation was observed between the FBTCS− and HC groups in the principal MS gradient differences and the PLS1 score map (*p*spin = 0.09). Between HC and FBTCS+ groups, a total of 5,553 genes were identified as significantly contributing to PLS1 (*p* < 0.005, BH-FDR correction), with 2,991 classified as PLS1 + and 2,562 as PLS1−. These gene sets were associated with the spatial pattern of principal MS gradient differences between HC and FBTCS+ groups.

Notably, the analysis was conducted exclusively for PLS1 − because the PLS1 + disease analysis did not yield significant results. The gene enrichment results for PLS1 + are shown in Supplementary Figures S1–S4. Figure 3D presents the results of epilepsy-related phenotype enrichment analysis based on PLS1 − gene list. The analysis revealed that absence seizures and visual seizures showed the highest levels of significance [*q* = 1 × 10^−12^, −log10(q) = 12]. Status epilepticus followed closely [*q* = 1 × 10^−11^, −log10(q) = 11]. Other phenotypes, including focal seizures [*q* = 1.25 × 10^−9^, −log10(q) = 9.1], tonic–clonic seizures [*q* = 2.51 × 10^−9^, −log10(q) = 8.6], complex partial seizures [*q* = 3.16 × 10^−9^, −log10(q) = 8.5], and clonic seizures [*q* = 1.26 × 10^−8^, −log10(q) = 7.9], also displayed significant enrichment. Additionally, temporal lobe epilepsy [*q* = 5.01 × 10^−7^, −log10(q) = 6.3] and generalized seizures [*q* = 6.31 × 10^−7^, −log10(q) = 6.2] showed moderate levels of enrichment.

### 3.4 Functional enrichment and upstream regulatory analyses of PLS1-associated genes

**Figure 4:**
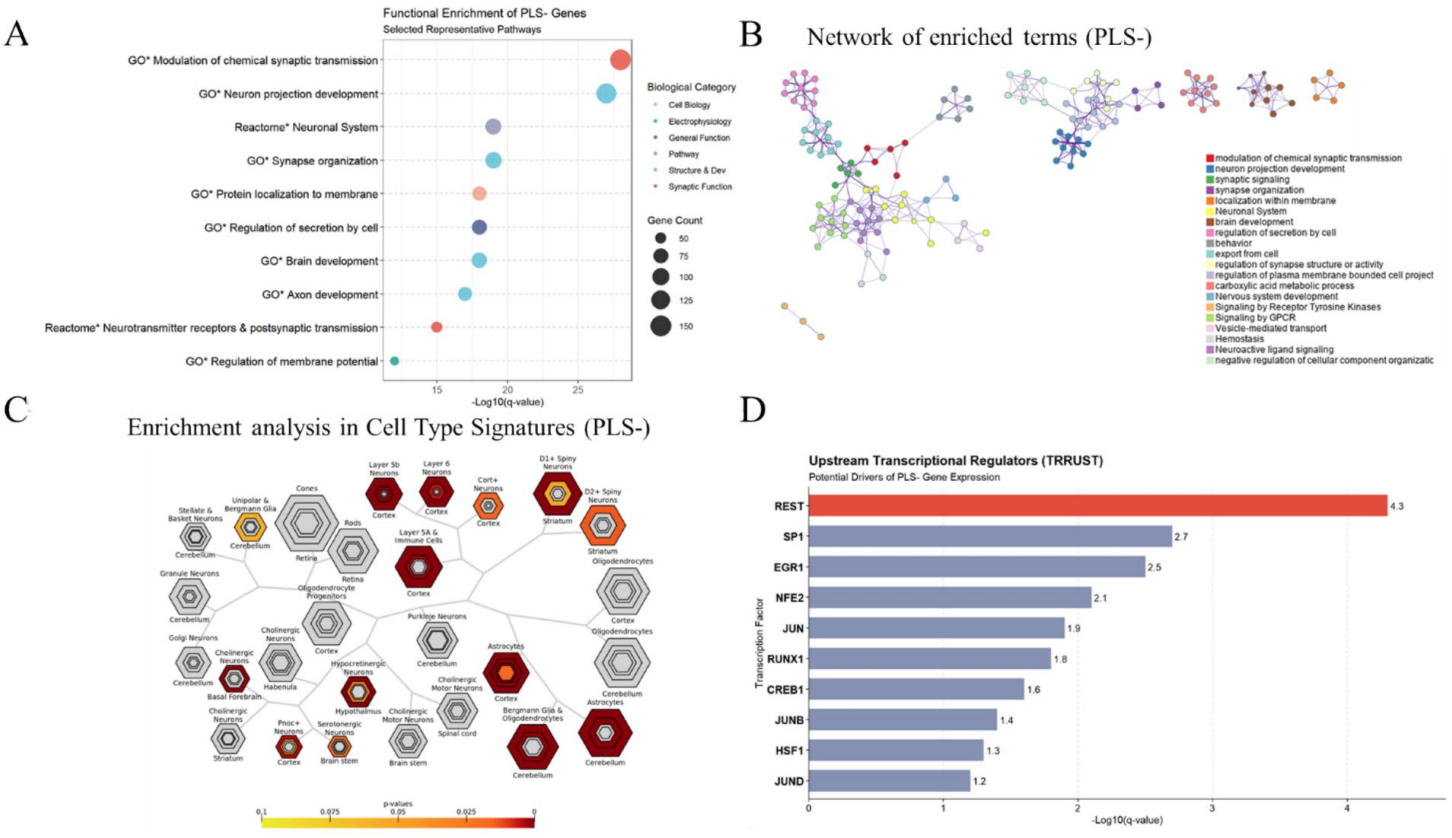
Functional enrichment and regulatory analysis. **(A)** The bubble chart illustrates the GO and Reactome functional annotations associated with PLS1 − genes. **(B)** The Metascape enrichment network visualization highlights both intra-cluster and inter-cluster similarities among enriched pathways. **(C)** The network of enrichment analysis in cell type signatures for PLS genes illustrates the distribution of PLS gene enrichment across various cell types. Each node represents a specific cell type, with the color intensity reflecting the significance of enrichment, where darker colors indicate higher significance. Connections between nodes reveal potential functional relationships among these cell types. **(D)** The analysis of upstream transcriptional regulators of PLS genes using TRRUST highlights the potential drivers of PLS gene expression. REST emerges as the most significant regulator, followed by SP1 and EGR1, suggesting their crucial roles in modulating PLS gene activity.

Gene function annotation was performed using Metascape, with a background set of 15,632 genes that have valid brain expression data. PLS1 − gene list was primarily enriched in several GO biological processes and Reactome pathways (Figures 4A,B). In contrast, functional enrichment analysis on the PLS1 + gene list did not reveal any significant disease-related enrichment. The detailed results of this analysis are provided in the Supplementary material (GO_DisGeNET). As such, we did not further expand on PLS1 + enrichment. Additional results from downstream analyses are also included in the supplementary files for reference. Furthermore, using CSEA, we investigated whether PLS1 − genes are enriched in specific human brain regions (Figure 4C). The results show that PLS1 − genes are significantly enriched in multiple specific brain cell types, particularly in hypothalamic Hyp. Hcrt cells (Hyp. Hcrt), where it is significantly enriched at a pSI threshold of 0.05 (*p* = 9.59 × 10^−5^, FDR-corrected *p* = 3.73 × 10^−4^). Additionally, Cb. Septin4 cell cluster (Cb. Septin4) are significantly enriched at a pSI threshold of 0.05 (*p* = 1.65 × 10^−10^, FDR-corrected *p* = 1.92 × 10^−9^), while Ctx. Fthfd cell cluster (Ctx. Fthfd) are significantly enriched at a pSI threshold of 0.001 (*p* = 2.60 × 10^−6^, FDR-corrected *p* = 9.10 × 10^−5^). These results suggest that the expression of PLS1 − genes is significantly enriched in specific cell types in the hypothalamus, cerebellum, and cortex, indicating non-random cellular enrichment patterns associated with the spatial transcriptomic signature of gradient alteration.

Through TRRUST analysis (Figure 4D), upstream transcriptional regulators potentially driving PLS1 gene expression were identified. REST exhibited the strongest regulatory influence, with the highest significance (*p* = 5.01 × 10^−5^). Other notable regulators included SP1 (*p* = 2.00 × 10^−3^), EGR1 (*p* = 3.16 × 10^−3^), and NFE2 (*p* = 7.94 × 10^−3^). Additionally, transcription factors such as JUN, RUNX1, and CREB1 demonstrated moderate regulatory potential, suggesting that these transcription factors may be involved in the molecular regulation underlying the observed imaging-transcriptomic associations.

## 4 Discussion

This study investigated alterations in cortical MS gradients in TLE patients with and without FBTCS. Our results suggested that, compared to healthy controls, FBTCS+ TLE patients exhibited significantly reduced principal MS gradients within the DMN. These gradient alterations showed significant spatial associations with gene expression patterns enriched for neurobiological pathways. In contrast, FBTCS− TLE patients did not show the DMN gradient abnormalities or region-specific changes observed in FBTCS+ patients, nor were significant correlations with gene expression detected. These findings suggest disrupted DMN gradients may be associated with the occurrence of FBTCS, which could provide a new perspective for understanding the structural and molecular basis underlying the heterogeneity of seizure symptoms.

In this study, compared to the HC group, FBTCS+ TLE patients exhibited significantly reduced principal MS gradient in the left inferior temporal gyrus (part 3). In contrast, increased principal MS gradients were observed in the left calcarine cortex (part 1) and right entorhinal cortex (part 1) in FBTCS+ TLE patients compared to the HC group. As a key hub within the medial temporal lobe, the entorhinal cortex plays a critical role in memory, spatial navigation, and seizure propagation in TLE (Feng et al., 2025; Schmidt et al., 2024). The increased MS gradients in the right entorhinal cortex observed in FBTCS+ TLE patients may reflect altered large-scale morphometric organization associated with seizure-related network vulnerability, aligning with its well-documented involvement in seizure dynamics and cognitive impairments. Similarly, increased MS gradients in the left calcarine cortex were observed in FBTCS+ TLE patients. As a region central to visual processing, its involvement may indicate broader network disruptions extending beyond the medial temporal lobe. Recent research has shown that spatial gradients of microstructural differentiation in TLE are disrupted, particularly in paralimbic regions, reflecting large-scale cortical reorganization and functional network impairments associated with cognitive dysfunction (Royer et al., 2023). Additionally, recent findings have revealed that atypical functional topographies and reduced differentiation between sensory and transmodal association cortices in TLE are linked to large-scale cortical reorganization and impaired functional signal flow, particularly affecting memory-related networks (Xie et al., 2024). These findings emphasize the entorhinal cortex’s pivotal role in seizure networks and suggest that extratemporal regions, such as the calcarine cortex, are also associated with the complex pathophysiology of FBTCS+ TLE. Notably, the left precuneus (part 2) also showed a significantly lower principal MS gradient in FBTCS+ patients compared to FBTCS− patients, which is one of the key structural gradient differences between the two subtypes. Furthermore, the principal MS gradient in the DMN was significantly altered in FBTCS+ patients, whereas no such DMN gradient alterations were found in FBTCS− patients, suggesting that DMN-related structural gradient disruption may be more characteristic of TLE patients with FBTCS. These DMN alterations may reflect the susceptibility of this network to recurrent epileptic activity originating from the temporal lobe and may be relevant to large-scale seizure generalization (Bu et al., 2024; Ishizaki et al., 2023). Furthermore, DMN gradient abnormalities may impair its interaction with other brain networks, contributing to the progression from focal to bilateral tonic–clonic seizures (Su et al., 2025). In contrast, the absence of DMN impairment in FBTCS− patients may be consistent with a more spatially restricted pattern of network involvement in FBTCS− patients and do not develop into bilateral tonic–clonic seizures. These findings may provide novel insights into the structural network abnormalities in TLE and the mechanisms underlying seizure heterogeneity.

Abnormal neuronal discharges and glial dysfunction may lead to neural network remodeling, dysregulated gene expression patterns, and imbalances in molecular signaling pathways, which may play a role in the onset and progression of epilepsy (Scheiblich et al., 2024). In this study, a significant positive correlation was observed between gene expression patterns and the gradient difference maps between patients and controls, and transcriptional signatures associated with gradient alterations in FBTCS+ TLE were identified. In contrast, no such significant correlation was found in FBTCS− patients, indicating that the association between MS network gradient alterations and specific gene expression patterns may indicate that imaging-transcriptomic coupling is more prominent in FBTCS+ TLE than in FBTCS− TLE. MS gradient alterations have been reported in major depressive disorder, highlighting molecular mechanisms of network changes, and similar approaches could illuminate the molecular basis of TLE-related gradient alterations observed in our study (Li et al., 2021; Xue et al., 2023). Similarly, recent studies using PET and fMRI data have shown that TLE-related network and metabolic changes are closely linked to gene expression patterns, further supporting the role of transcriptional dysregulation in TLE (Hu et al., 2025). Another study demonstrated that altered brain network dynamics and metabolic processes in TLE patients are associated with specific gene expression profiles, further highlighting the interplay between molecular signaling and structural network changes in epilepsy (Ran et al., 2025). Our findings are generally consistent with previous studies suggesting that abnormal gene expression mediates the remodeling of structural networks and further support the relevance of spatially specific gene expression patterns to TLE-related network abnormalities (Chen et al., 2025).

Our enrichment analysis results indicated that PLS1 − genes were significantly enriched in absence seizures, visual seizures, status epilepticus, and tonic–clonic seizures, indicating overlap with molecular signatures previously linked to multiple seizure phenotypes. Specifically, the enrichment observed highlights potential molecular mechanisms related to the extensive cortical involvement and heightened neuronal hyperexcitability that characterize TLE patients with FBTCS. Given the clinical relevance of FBTCS as a marker of seizure severity and its association with widespread structural and functional network disruptions, the identified gene dysregulation may also reflect altered connectivity gradients and cortical remodeling processes that exacerbate seizure propagation and severity. This finding aligns with large-scale studies on epilepsy-related gene expression patterns, which revealed shared molecular mechanisms across seizure types and their association with structural network alterations (Larivière et al., 2022). The dysregulation of these genes may be related to MS network gradient abnormalities through their influence on cortical morphometric features, consistent with earlier research linking gene expression to structural network remodeling in epilepsy (Yin et al., 2023). Such hierarchical network disruptions have also been observed in atypical connectivity gradients in specific epilepsy syndromes, highlighting their role in clinical symptom variability and cognitive impairments (Zhang et al., 2023). This remodeling may be relevant to the network architecture associated with seizure generalization and may help to explain the clinical symptom variability and cognitive impairments observed in FBTCS+ TLE patients (Pan et al., 2021), whereas the absence of comparable findings in FBTCS− patients may suggest a less extensive degree of network-level alteration.

TLE is widely considered to result from alterations in neuronal excitability, with changes in chemical and electrical communication between cells being one of the primary causes. Glutamate and GABA receptor proteins, composed of ionotropic ligand-gated ion channels and metabotropic G-protein-coupled receptor subfamilies, play a crucial role in the fine-tuned regulation of neural networks. Dysfunction in these receptors can lead to an excitatory-inhibitory imbalance, which is a key factor in epilepsy pathogenesis (Ren and Curia, 2021). Functional enrichment analysis further suggested that PLS1 − genes were mainly enriched in pathways related to synaptic transmission, regulation of neuronal excitability, neurodevelopment, and intercellular signaling. These findings are consistent with prior studies highlighting the role of these processes in epilepsy pathophysiology and network alterations (Abudusalamu et al., 2025). Specifically, pathways such as modulation of chemical synaptic transmission, the reactive neuronal system, and synapse organization are enriched, underscoring their importance in maintaining proper neuronal communication. Disruptions in these processes may contribute to the neuronal hyperexcitability observed in TLE. Recent studies using MS network analysis in mTLE have linked structural alterations to neuroinflammatory gene expression, particularly in patients with hippocampal sclerosis, implicating pathways related to neurodevelopment and neurodegeneration in epilepsy progression (Lu et al., 2025). These findings suggest that the transcriptomic signature linked to gradient alteration is related to biological processes relevant to neuronal communication and excitability. Dysregulation of synaptic and excitability-related pathways has been implicated in seizure propagation and clinical severity in previous studies (Liu et al., 2024).

In addition, TRRUST analysis identified REST as a key transcriptional repressor significantly upregulated in seizure-related molecular responses, particularly in TLE patients with FBTCS. Dysregulation of REST has been implicated in increased neuronal excitability, pathological network remodeling, and heightened neuronal and glial cell death (Lim et al., 2025). By repressing genes critical for synaptic plasticity and excitatory-inhibitory balance, REST may exacerbate maladaptive processes that destabilize neural circuits and promote seizure recurrence. These molecular and cellular changes are closely linked to disruptions in large-scale functional networks including the DMN (Garcia-Manteiga et al., 2019; Roopra et al., 2012). SP1 and EGR1, which are closely associated with neurodevelopment, synaptic plasticity, and stress responses, may be involved in molecular pathways related to network remodeling and epileptic pathology through their dysregulated activity and expression (Zhao et al., 2020; Dong et al., 2023). The abnormal regulation of these transcription factors may not only directly affect downstream target gene expression but may also alter the morphometric and functional states of neural cells, potentially linked to the observed MS gradient alterations (Sattarifard et al., 2023).

This study has several limitations. First, we utilized only five cortical morphometric features to construct the MS gradients, which might limit the comprehensive characterization of brain structural properties. For FBTCS− patients, who showed less pronounced gradient abnormalities in the present study, more diverse morphometric features may be needed to detect subtle gradient alterations and their potential molecular correlates that were not identified in this study. Although the use of morphometric features to construct network gradients has been widely adopted in brain network studies, future research could integrate additional morphometric or functional features to provide a more holistic representation of brain networks. Second, the sample size in our study is relatively small. While comparable to existing studies on TLE, larger cohorts are needed to improve statistical power, validate the reproducibility of MS gradient findings, and better characterize between-subtype heterogeneity in TLE. Third, differences in clinical variables, such as age of onset and disease duration, may reflect more severe neural network abnormalities in FBTCS+ patients, and future studies could further explore these mechanisms. In addition, the recruitment of patients and the control of confounding variables remain challenging, which may impact the interpretation of results. Moreover, as with previous studies using the AHBA dataset, the exclusion of right hemisphere data due to limited availability remains a limitation that may hinder the understanding of bilateral processes. Future studies utilizing more comprehensive transcriptomic datasets could address this gap and provide deeper insights into the molecular mechanisms and whole-brain effects of TLE.

This study identified altered cortical MS gradient organization in TLE, with more prominent network-level and imaging-transcriptomic abnormalities in FBTCS+ patients. By integrating morphometric gradient analysis with transcriptomic data, the study provides multiscale insight into the structural network alterations associated with FBTCS in TLE.

## Data Availability

The original contributions presented in the study are included in the article/Supplementary material. Further inquiries can be directed to the corresponding authors.

## Footnotes

1
http://human.brain-map.org

2
https://github.com/rmarkello/abagen

3
https://metascape.org

4
https://sites.wustl.edu

## References

[ref1] Abdi H. Williams L. J. (2013). Partial least squares methods: partial least squares correlation and partial least square regression. Methods Mol. Biol. 930, 549–579. doi: 10.1007/978-1-62703-059-5_23, 23086857

[ref2] Abudusalamu R. Maimaiti A. Han W. Wang X. Jiao T. Han D. . (2025). Genetic relationship between epilepsy and mental disorders: a comprehensive GWAS analysis. Epilepsy Behav. 171:110500. doi: 10.1016/j.yebeh.2025.110500, 40460570

[ref3] Arnatkeviciute A. Fulcher B. D. Fornito A. (2019). A practical guide to linking brain-wide gene expression and neuroimaging data. NeuroImage 189, 353–367. doi: 10.1016/j.neuroimage.2019.01.011, 30648605

[ref4] Blumenfeld H. Varghese G. I. Purcaro M. J. Motelow J. E. Enev M. McNally K. A. . (2009). Cortical and subcortical networks in human secondarily generalized tonic-clonic seizures. Brain 132, 999–1012. doi: 10.1093/brain/awp028, 19339252 PMC2724910

[ref5] Bu J. Yin H. Ren N. Zhu H. Xu H. Zhang R. . (2024). Structural and functional changes in the default mode network in drug-resistant epilepsy. Epilepsy Behav. 151:109593. doi: 10.1016/j.yebeh.2023.109593, 38157823

[ref6] Burianová H. Faizo N. L. Gray M. Hocking J. Galloway G. Reutens D. (2017). Altered functional connectivity in mesial temporal lobe epilepsy. Epilepsy Res. 137, 45–52. doi: 10.1016/j.eplepsyres.2017.09.001, 28923408

[ref7] Chen X. Zhang X. Su S. Zhou Q. Qin B. Fan L. . (2025). Potential molecular mechanisms explaining progressive alterations of the structural network in temporal lobe epilepsy. Neurobiol. Dis. 215:107092. doi: 10.1016/j.nbd.2025.107092, 40930426

[ref8] Crow A. J. D. Thomas A. Rao Y. Beloor-Suresh A. Weinstein D. Hinds W. A. . (2023). Task-based functional magnetic resonance imaging prediction of postsurgical cognitive outcomes in temporal lobe epilepsy: a systematic review, meta-analysis, and new data. Epilepsia 64, 266–283. doi: 10.1111/epi.17475, 36522799 PMC9944224

[ref9] Desikan R. S. Ségonne F. Fischl B. Quinn B. T. Dickerson B. C. Blacker D. . (2006). An automated labeling system for subdividing the human cerebral cortex on MRI scans into gyral based regions of interest. NeuroImage 31, 968–980. doi: 10.1016/j.neuroimage.2006.01.021, 16530430

[ref10] Dong Z. Min F. Zhang S. Zhang H. Zeng T. (2023). EGR1-driven METTL3 activation curtails VIM-mediated neuron injury in epilepsy. Neurochem. Res. 48, 3349–3362. doi: 10.1007/s11064-023-03950-8, 37268752

[ref11] Dougherty J. D. Schmidt E. F. Nakajima M. Heintz N. (2010). Analytical approaches to RNA profiling data for the identification of genes enriched in specific cells. Nucleic Acids Res. 38, 4218–4230. doi: 10.1093/nar/gkq130, 20308160 PMC2910036

[ref12] Ellsay A. C. Winston G. P. (2024). Advances in MRI-based diagnosis of temporal lobe epilepsy: correlating hippocampal subfield volumes with histopathology. J. Neuroimaging 34, 515–526. doi: 10.1111/jon.13225, 39092876

[ref13] Feng Y. Diego K. S. Dong Z. Christenson Wick Z. Page-Harley L. Page-Harley V. . (2025). Distinct changes to hippocampal and medial entorhinal circuits emerge across the progression of cognitive deficits in epilepsy. Cell Rep. 44:115131. doi: 10.1016/j.celrep.2024.115131, 39847482 PMC11949077

[ref14] Fonseca E. Sarria-Estrada S. Pareto D. Turon M. Quintana M. Santamarina E. . (2023). Relationship between visuoperceptual functions and parietal structural abnormalities in temporal lobe epilepsy. Brain Imaging Behav. 17, 35–43. doi: 10.1007/s11682-022-00738-2, 36357555

[ref15] Fulcher B. D. Arnatkeviciute A. Fornito A. (2021). Overcoming false-positive gene-category enrichment in the analysis of spatially resolved transcriptomic brain atlas data. Nat. Commun. 12:2669. doi: 10.1038/s41467-021-22862-1, 33976144 PMC8113439

[ref16] Garcia-Manteiga J. M. D’Alessandro R. Meldolesi J. (2019). News about the role of the transcription factor REST in neurons: from physiology to pathology. Int. J. Mol. Sci. 21:235. doi: 10.3390/ijms21010235, 31905747 PMC6982158

[ref17] Ge Y. Chen C. Li H. Wang R. Yang Y. Ye L. . (2024). Altered structural network in temporal lobe epilepsy with focal to bilateral tonic-clonic seizures. Ann. Clin. Transl. Neurol. 11, 2277–2288. doi: 10.1002/acn3.52135, 39152643 PMC11537139

[ref18] Hawrylycz M. J. Lein E. S. Guillozet-Bongaarts A. L. Shen E. H. Ng L. Miller J. A. . (2012). An anatomically comprehensive atlas of the adult human brain transcriptome. Nature 489, 391–399. doi: 10.1038/nature11405, 22996553 PMC4243026

[ref19] Hu J. Cui B. Wang Z. Wang J. Xu X. Lu J. (2025). Transcriptomic and glucose metabolism of connectome dynamics variability in temporal lobe epilepsy revealed by simultaneous PET-fMRI. Neurobiol. Dis. 212:106967. doi: 10.1016/j.nbd.2025.106967, 40398518

[ref20] Ishizaki T. Maesawa S. Nakatsubo D. Yamamoto H. Torii J. Mutoh M. . (2023). Connectivity alteration in thalamic nuclei and default mode network-related area in memory processes in mesial temporal lobe epilepsy using magnetoencephalography. Sci. Rep. 13:10632. doi: 10.1038/s41598-023-37834-2, 37391474 PMC10313774

[ref21] Janszky J. Janszky I. Schulz R. Hoppe M. Behne F. Pannek H. W. . (2005). Temporal lobe epilepsy with hippocampal sclerosis: predictors for long-term surgical outcome. Brain 128, 395–404. doi: 10.1093/brain/awh358 15634733

[ref22] Larivière S. Royer J. Rodríguez-Cruces R. Paquola C. Caligiuri M. E. Gambardella A. . (2022). Structural network alterations in focal and generalized epilepsy assessed in a worldwide ENIGMA study follow axes of epilepsy risk gene expression. Nat. Commun. 13:4320. doi: 10.1038/s41467-022-31730-5, 35896547 PMC9329287

[ref23] Leyden K. M. Kucukboyaci N. E. Puckett O. K. Lee D. Loi R. Q. Paul B. . (2015). What does diffusion tensor imaging (DTI) tell us about cognitive networks in temporal lobe epilepsy? Quant. Imaging Med. Surg. 5, 247–263. doi: 10.3978/j.issn.2223-4292.2015.02.01, 25853083 PMC4379319

[ref24] Li J. Seidlitz J. Suckling J. Fan F. Ji G. J. Meng Y. . (2021). Cortical structural differences in major depressive disorder correlate with cell type-specific transcriptional signatures. Nat. Commun. 12:1647. doi: 10.1038/s41467-021-21943-5, 33712584 PMC7955076

[ref25] Lim C. T. Lim C. W. Huang T. Ismail E. N. Reisi P. Cheah P. S. . (2025). The regulatory roles of REST in the synaptic development, function and related neurological disorders. J. Neurochem. 169:e70132. doi: 10.1111/jnc.70132, 40534452

[ref26] Liu Q. Shen C. Dai Y. Tang T. Hou C. Yang H. . (2024). Single-cell, single-nucleus and xenium-based spatial transcriptomics analyses reveal inflammatory activation and altered cell interactions in the hippocampus in mice with temporal lobe epilepsy. Biomark. Res. 12:103. doi: 10.1186/s40364-024-00636-3, 39272194 PMC11396644

[ref27] Liu C. Zhang X. Nguyen T. T. Liu J. Wu T. Lee E. . (2022). Partial least squares regression and principal component analysis: similarity and differences between two popular variable reduction approaches. Gen Psychiatr 35:e100662. doi: 10.1136/gpsych-2021-100662, 35146334 PMC8796256

[ref28] Long J. Li J. Xie B. Jiao Z. Shen G. Liao W. . (2023). Morphometric similarity network alterations in COVID-19 survivors correlate with behavioral features and transcriptional signatures. Neuroimage Clin 39:103498. doi: 10.1016/j.nicl.2023.103498, 37643521 PMC10474075

[ref29] Lu L. Zhao C. Liao W. Wang P. Zhang Y. An D. . (2025). Alternations in morphometric similarity network in mesial temporal epilepsy correlate to neuroinflammatory pathway gene transcriptions. Acta Epileptol 7:18. doi: 10.1186/s42494-025-00208-4, 40217352 PMC11960352

[ref30] Lucas A. Mouchtaris S. Cornblath E. J. Sinha N. Caciagli L. Hadar P. . (2023). Subcortical functional connectivity gradients in temporal lobe epilepsy. Neuroimage Clin 38:103418. doi: 10.1016/j.nicl.2023.103418, 37187042 PMC10196948

[ref31] Lynall M.-E. Bassett D. S. Kerwin R. McKenna P. J. Kitzbichler M. Muller U. . (2010). Functional connectivity and brain networks in schizophrenia. J. Neurosci. 30, 9477–9487. doi: 10.1523/JNEUROSCI.0333-10.2010, 20631176 PMC2914251

[ref32] Markello R. D. Arnatkeviciute A. Poline J. B. Fulcher B. D. Fornito A. Misic B. (2021). Standardizing workflows in imaging transcriptomics with the abagen toolbox. eLife 10:e72129. doi: 10.7554/eLife.72129, 34783653 PMC8660024

[ref33] Mueller S. G. Laxer K. D. Scanlon C. Garcia P. McMullen W. J. Loring D. W. . (2012). Different structural correlates for verbal memory impairment in temporal lobe epilepsy with and without mesial temporal lobe sclerosis. Hum. Brain Mapp. 33, 489–499. doi: 10.1002/hbm.21226, 21438080 PMC3259857

[ref34] Pan L. Wu Y. Bao J. Guo D. Zhang X. Wang J. . (2021). Alterations in neural networks during working memory encoding related to cognitive impairment in temporal lobe epilepsy. Front. Hum. Neurosci. 15:770678. doi: 10.3389/fnhum.2021.770678, 35069151 PMC8766724

[ref35] Pizzanelli C. Pesaresi I. Milano C. Cecchi P. Fontanelli L. Giannoni S. . (2022). Distinct limbic connectivity in left and right benign mesial temporal lobe epilepsy: evidence from a resting state functional MRI study. Front. Neurol. 13:943660. doi: 10.3389/fneur.2022.943660, 36247782 PMC9558280

[ref36] Prevey M. L. Delaney R. C. Cramer J. A. Mattson R. H. (1998). Complex partial and secondarily generalized seizure patients: cognitive functioning prior to treatment with antiepileptic medication. VA epilepsy cooperative study 264 group. Epilepsy Res. 30, 1–9. doi: 10.1016/S0920-1211(97)00091-0, 9551840

[ref37] Qin L. Zhou Q. Sun Y. Pang X. Chen Z. Zheng J. (2024). Dynamic functional connectivity and gene expression correlates in temporal lobe epilepsy: insights from hidden markov models. J. Transl. Med. 22:763. doi: 10.1186/s12967-024-05580-2, 39143498 PMC11323657

[ref38] Qu J. Qu Y. Zhu R. Wu Y. Xu G. Wang D. (2024). Transcriptional expression patterns of the cortical morphometric similarity network in progressive supranuclear palsy. CNS Neurosci. Ther. 30:e14901. doi: 10.1111/cns.14901, 39097922 PMC11298202

[ref39] Ran H. Yu Q. Hu Y. Cui J. Huang K. Xie Y. . (2025). Alterations of multilayer network correlated with cognitive impairment and gene expression profiles in children with idiopathic generalized epilepsy. Sci. Rep. 15:36877. doi: 10.1038/s41598-025-20784-2, 41125675 PMC12546745

[ref40] Ren E. Curia G. (2021). Synaptic reshaping and neuronal outcomes in the temporal lobe epilepsy. Int. J. Mol. Sci. 22:3860. doi: 10.3390/ijms22083860, 33917911 PMC8068229

[ref41] Roopra A. Dingledine R. Hsieh J. (2012). Epigenetics and epilepsy. Epilepsia 53, 2–10. doi: 10.1111/epi.12030, 23216574 PMC3531878

[ref42] Royer J. Larivière S. Rodriguez-Cruces R. Cabalo D. G. Tavakol S. Auer H. . (2023). Cortical microstructural gradients capture memory network reorganization in temporal lobe epilepsy. Brain 146, 3923–3937. doi: 10.1093/brain/awad125, 37082950 PMC10473569

[ref43] Sadikov A. Choi H. L. Cai L. T. Mukherjee P. (2025). Estimating brain similarity networks with diffusion MRI. Hum. Brain Mapp. 46:e70313. doi: 10.1002/hbm.70313, 40782044 PMC12335008

[ref44] Sattarifard H. Safaei A. Khazeeva E. Rastegar M. Davie J. R. (2023). Mitogen and stress-activated protein kinase (MSK1/2) regulated gene expression in normal and disease states. Biochem. Cell Biol. 101, 204–219. doi: 10.1139/bcb-2022-0371, 36812480

[ref45] Scheiblich H. Eikens F. Wischhof L. Opitz S. Jüngling K. Cserép C. . (2024). Microglia rescue neurons from aggregate-induced neuronal dysfunction and death through tunneling nanotubes. Neuron 112, 3106–3125. doi: 10.1016/j.neuron.2024.06.029, 39059388

[ref46] Schmidt M. Bauer T. Kehl M. Minarik A. Walger L. Schultz J. . (2024). Olfactory dysfunction and limbic Hypoactivation in temporal lobe epilepsy. Hum. Brain Mapp. 45:e70061. doi: 10.1002/hbm.70061, 39487626 PMC11530705

[ref47] Seidlitz J. Váša F. Shinn M. Romero-Garcia R. Whitaker K. J. Vértes P. E. . (2018). Morphometric similarity networks detect microscale cortical organization and predict inter-individual cognitive variation. Neuron 97, 231–247.e7. doi: 10.1016/j.neuron.2017.11.039, 29276055 PMC5763517

[ref48] Su S. Zhou Q. Chen X. Qin B. Sun Y. Qin L. . (2025). Changes in cerebellar functional gradients and molecular genetic mechanisms in patients with temporal lobe epilepsy: a cross-sectional and longitudinal functional magnetic resonance imaging study. Cerebellum (London, England) 24:155. doi: 10.1007/s12311-025-01900-4 40956365

[ref49] Tabibian F. Mehvari Habibabadi J. Maracy M. R. Kahnouji H. Rahimi M. Rezaei M. (2023). Evaluation of cognitive impairment in refractory temporal lobe epilepsy patients concerning structural brain lesions. Basic Clin Neurosci 14, 385–396. doi: 10.32598/bcn.2022.3827.1, 38077172 PMC10700814

[ref50] Wirrell E. C. Nabbout R. Scheffer I. E. Alsaadi T. Bogacz A. French J. A. . (2022). Methodology for classification and definition of epilepsy syndromes with list of syndromes: report of the ILAE task force on nosology and definitions. Epilepsia 63, 1333–1348. doi: 10.1111/epi.17237, 35503715

[ref51] Xie K. Royer J. Larivière S. Rodriguez-Cruces R. Frässle S. Cabalo D. G. . (2024). Atypical connectome topography and signal flow in temporal lobe epilepsy. Prog. Neurobiol. 236:102604. doi: 10.1016/j.pneurobio.2024.102604, 38604584

[ref52] Xue K. Guo L. Zhu W. Liang S. Xu Q. Ma L. . (2023). Transcriptional signatures of the cortical morphometric similarity network gradient in first-episode, treatment-naive major depressive disorder. Neuropsychopharmacology 48, 518–528. doi: 10.1038/s41386-022-01474-3, 36253546 PMC9852427

[ref53] Yakovleva K. D. Dmitrenko D. V. Panina I. S. Usoltseva A. A. Gazenkampf K. A. Konovalenko O. V. . (2022). Expression profile of miRs in mesial temporal lobe epilepsy: systematic review. Int. J. Mol. Sci. 23:951. doi: 10.3390/ijms23020951, 35055144 PMC8781102

[ref54] Yang S. Wagstyl K. Meng Y. Zhao X. Li J. Zhong P. . (2021). Cortical patterning of morphometric similarity gradient reveals diverged hierarchical organization in sensory-motor cortices. Cell Rep. 36:109582. doi: 10.1016/j.celrep.2021.109582, 34433023

[ref55] Yao G. Luo J. Zou T. Li J. Hu S. Yang L. . (2024). Transcriptional patterns of the cortical morphometric inverse divergence in first-episode, treatment-naïve early-onset schizophrenia. NeuroImage 285:120493. doi: 10.1016/j.neuroimage.2023.120493, 38086496

[ref56] Yeo B. T. Krienen F. M. Sepulcre J. Sabuncu M. R. Lashkari D. Hollinshead M. . (2011). The organization of the human cerebral cortex estimated by intrinsic functional connectivity. J. Neurophysiol. 106, 1125–1165. doi: 10.1152/jn.00338.2011, 21653723 PMC3174820

[ref57] Yin Y. Wang F. Ma Y. Yang J. Li R. Li Y. . (2023). Structural and functional changes in drug-naïve benign childhood epilepsy with centrotemporal spikes and their associated gene expression profiles. Cereb. Cortex 33, 5774–5782. doi: 10.1093/cercor/bhac458, 36444721 PMC10183734

[ref58] Yin X. Yang J. Xiang Q. Peng L. Song J. Liang S. . (2024). Brain network hierarchy reorganization in subthreshold depression. Neuroimage Clin 42:103594. doi: 10.1016/j.nicl.2024.103594, 38518552 PMC10973537

[ref59] Zaitsev A. V. Khazipov R. (2023). Molecular and cellular mechanisms of epilepsy. Int. J. Mol. Sci. 24:12415. doi: 10.3390/ijms241512415, 37569790 PMC10418982

[ref60] Zeng T. Wang X. Zhang Y. Mao M. Hong C. Yu Z. . (2026). Neuroendocrine-vascular interaction is associated with thalamocortical disorganization and cognitive decline in perimenopausal hypertension. Front. Med. 13:1775371. doi: 10.3389/fmed.2026.1775371, 41836925 PMC12983412

[ref61] Zhang Q. Li J. He Y. Yang F. Xu Q. Larivière S. . (2023). Atypical functional connectivity hierarchy in Rolandic epilepsy. Commun Biol 6:704. doi: 10.1038/s42003-023-05075-8, 37429897 PMC10333191

[ref62] Zhang P.-P. Li M. S. Zhou J. Zhu C. H. Tang R. He Z. C. . (2026). Region-resolved proteomic map of the human brain: functional interconnections and neurological implications. Signal Transduct. Target. Ther. 11:43. doi: 10.1038/s41392-025-02554-8, 41633974 PMC12868004

[ref63] Zhao M.-W. Qiu W.-J. Yang P. (2020). SP1 activated-lncRNA SNHG1 mediates the development of epilepsy via miR-154-5p/TLR5 axis. Epilepsy Res. 168:106476. doi: 10.1016/j.eplepsyres.2020.106476, 33096314

[ref64] Zhou Y. Zhou B. Pache L. Chang M. Khodabakhshi A. H. Tanaseichuk O. . (2019). Metascape provides a biologist-oriented resource for the analysis of systems-level datasets. Nat. Commun. 10:1523. doi: 10.1038/s41467-019-09234-6, 30944313 PMC6447622

