# Supplementary material for: Transcriptional signatures of the cortical morphometric similarity network gradient in left temporal lobe epilepsy with different seizure symptoms
Source: Front Neurosci. 2026 Jun 3;20:1833695. doi: 10.3389/fnins.2026.1833695 (PMC13273452; doi:10.3389/fnins.2026.1833695)
Supplement: Supplementary file 2 [file Data_Sheet_2.docx]

**Gene enrichment results for PLS1+**


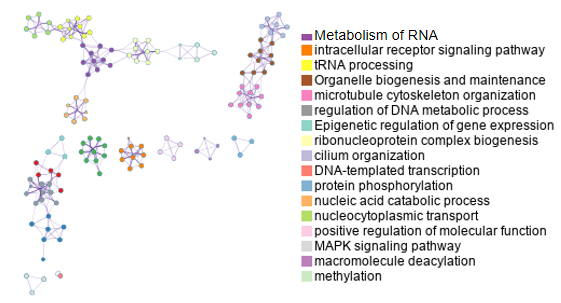


**Supplementary Figure 1 Functional enrichment network of GO terms between FBTCS+ and HC groups (PLS+).**


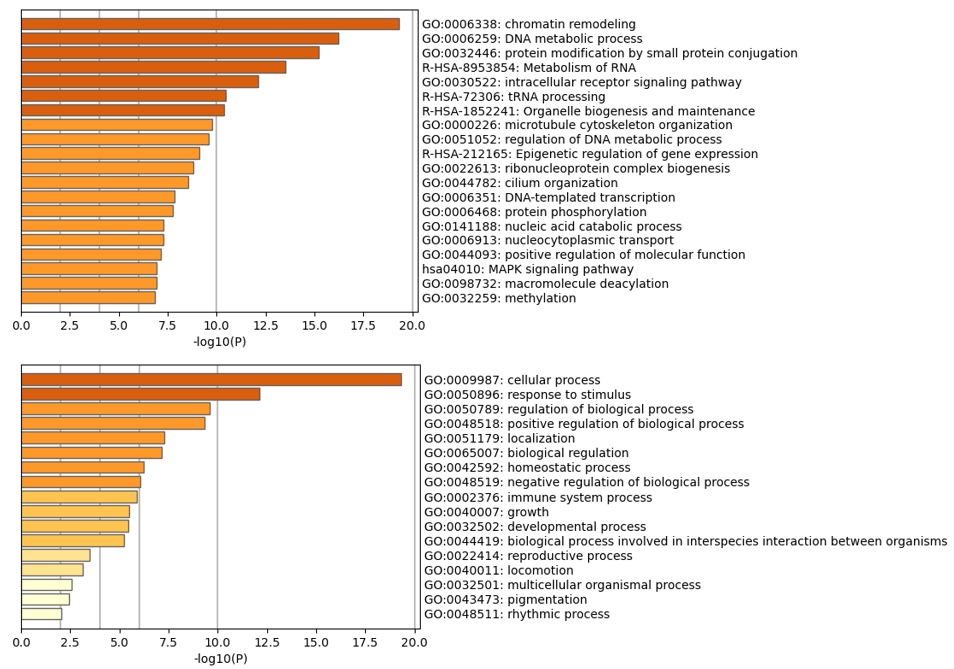


**Supplementary Figure 2 Functional enrichment analysis of GO terms and their parent categories between FBTCS+ and HC groups (PLS+). A.** Selected GO Terms **B.** Parent GO Categories


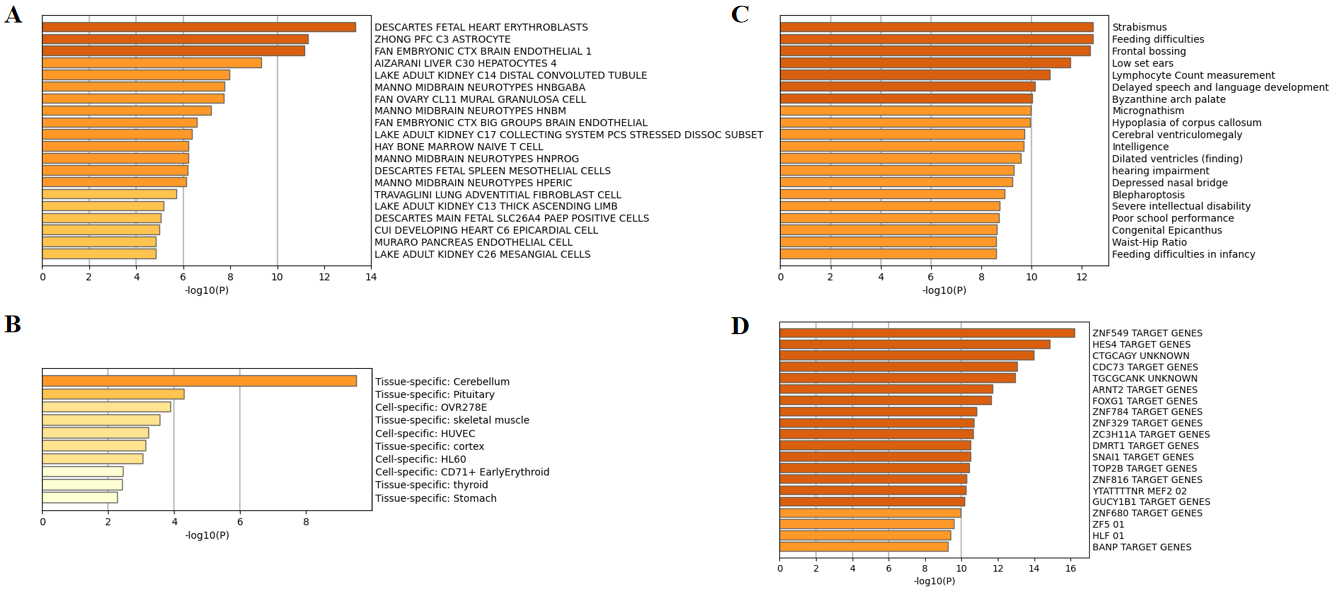


**Supplementary Figure 3 Functional enrichment and regulatory analysis between FBTCS+ and HC groups (PLS+). A. C**ell Type Signatures Analysis **B.** DisGeNET-Based Enrichment Analysis **C.** PaGenBase Tissue Expression Analysis **D.** Transcription Factor Targets Analysis

**
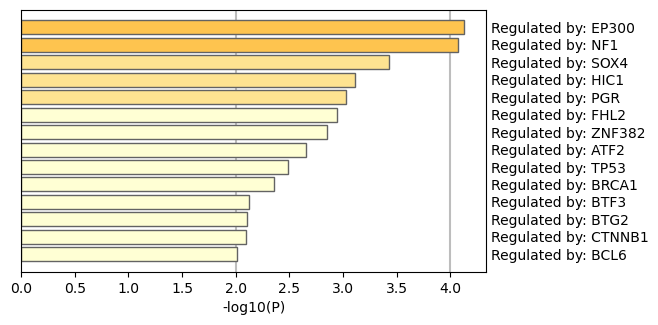
**

**Supplementary Figure 4 Transcription factor enrichment analysis based on TRRUST database between FBTCS+ and HC groups (PLS+).**
